# Conservative tissue sparing management of pediatric pyogenic granuloma with preserved joint function: A narrative review with an illustrative case

**DOI:** 10.1016/j.jpra.2026.01.043

**Published:** 2026-01-31

**Authors:** Alaa A. Sultan, Faris A. Sultan, Maha S. AlQahtani, Muhanad Alzahrani, Abdullah Alabbasi

**Affiliations:** a Division of Plastic Surgery, Department of Surgery, Faculty of Medicine, Umm Al-Qura University, Makkah, Saudi Arabia; b Department of Surgery, College of Medicine King Saud University, Riyadh, Saudi Arabia; c College of Medicine King, Saud University, Riyadh, Saudi Arabia; d Family Medicine, Ministry of Health, Jeddah, Saudi Arabia; e Saudi Board in Family Medicine, Ministry of Health, Jeddah, Saudi Arabia

**Keywords:** Pyogenic and lobular capillary hemangioma, Pediatric dermatology, Topical corticosteroids, Conservative treatment, Digital lesions, Contracture prevention

## Abstract

The pediatric population presents specific management challenges with pyogenic granuloma (PG), particularly when located near joints, as surgical treatment may compromise mobility. While surgical excision remains the gold standard treatment, emerging reports suggest that more conservative approaches may be suitable in select cases, offering less invasive treatment options. We report the preservation of joint function while successfully resolving pyogenic digital granuloma with topical steroid-antibiotic preparations. A healthy 7-year-old girl presented with progressive left middle and ring finger exacerbation of lesions attributable to sheep fence lacerations. Primary closure was performed; however, it resulted in recurrent serous collections, necessitating the use of a patch of granulation tissue. Examination revealed non-tender digit swelling at the volar aspect of the middle and distal phalanges with maintained flexor digitorum muscles, intact sensation, and no Kanavel signs. The child underwent staged bedside debridement under a digital block and removal of sutures lodged within the granulation tissue. Conservative treatment included daily steroid-infused dressings with fusidic acid ointment, overlapping two debridement sessions spaced 1 week apart. No lesions were noted post-treatment, along with a full range of movement and intact sensation. Skin loss traversing the flexor creases did not result in any form of flexion contraction and remained satisfactory at follow-up. This case demonstrates that dual-action corticosteroid-antibiotic therapy, combined with stepwise debridement, can achieve functional preservation of the joint in children with digital PG. In certain pediatric patients, where the lesions are located in functionally important regions, this surgical excision alternative is beneficial. It must be noted, however, that the lack of confirmation through histopathological analysis in this case represents a significant limitation that should be considered in the course of clinical reasoning.

## Introduction

Pyogenic granuloma (PG), also known as lobular capillary hemangioma (LCH), is a common, acquired, benign vascular proliferation of the skin and mucous membranes that was first described in 1897.^1^^,^^2^ Recently, PG has been reclassified as a capillary hemangioma and renamed LCH.^3^ This poses a clinical challenge owing to its uncertain etiology and treatment options.

Epidemiological data indicate that PG prevalence is observed across all age groups, spanning diverse anatomical locations, and exhibits distinct demographic patterns with various associations^1^^.^ The head and neck area is the most commonly affected site (56%), followed by the upper limbs (22%), trunk (16%), and lower limbs (6%).^3^ It can be idiopathic or secondary to trauma, underlying vascular malformations, infections, physiological or pathological endocrine changes, and hormone therapy.^4^

Histologically, LCH (previously termed PG) features lobular aggregates of capillary-sized vessels within the fibromyxoid stroma and mild inflammatory infiltrates. The lesion often appears as a well-circumscribed exophytic mass with overlying epidermal thinning, erosion, or ulceration. Deeper areas show increased vascular cellularity and occasional mitotic activity.^5^

Several factors must be considered when selecting an appropriate treatment for pediatric PG. The location and size of the lesion necessitate approaches that prioritize favorable cosmetic outcomes to minimize the scarring. Minimally invasive techniques are generally preferred in children to reduce procedural anxiety and discomfort in the future. Complete lesion removal is essential to reduce the risk of recurrence.^6^

The management of pediatric pyogenic granuloma involving functionally critical areas such as the digits remains challenging, particularly when lesions cross flexion creases or are adjacent to joints. While surgical excision is considered definitive, concerns regarding scarring, joint stiffness, and the need for anesthesia have prompted interest in less invasive strategies. This report describes a pediatric case of digital pyogenic granuloma managed with staged bedside debridement and topical corticosteroid–antibiotic therapy, focusing on clinical decision-making, functional outcomes, and limitations of conservative management.

## Case presentation

This case report presents a 7-year-old girl, medically and surgically free, who presented with a 2-week history of worsening wounds on the left middle and ring fingers. After suffering lacerations from sheep fencing, the patient was initially taken to a primary health clinic in their village. The wound was closed primarily under local anesthesia, and the patient was administered a 1-week course of oral antibiotics. A week later, the wounds began to develop small fluid collections. The patient was taken to the village clinic, the collections were drained using a needle, and the patient was administered another course of antibiotics. The family decided to bring the patient to the emergency department of King Khaled University Hospital in Riyadh when the collection recurred and worsened.

Upon presentation to the Emergency Department (ED), the patient had non-tender swellings on the left middle and ring fingers over the volar aspect of the middle and distal phalanges. The middle finger was red, massively swollen, and appeared to have a fluctuating collection with some discharge upon palpation. The swelling on the ring finger was hard and did not appear to contain fluid. The range of motion appeared to be normal, with intact FDS. Although the FDP was initially challenging to assess, it was confirmed to be intact after the injection of a local anesthetic. Vascularity and sensation were intact distally, and Kanavel signs were negative.

The patient underwent two sessions of bedside debridement of the wound under a digital block. The first session of bedside debridement was conducted in the emergency room under a digital block. Sutures from the initial repair were found deep within the granulation tissue and were removed. A second bedside debridement session was performed a week later, once the initial swelling had subsided, and the patient was kept on daily dressings with cortisone-containing fusidic acid ointment. No tissue specimens were submitted for histopathological examination, as the procedure involved bedside debridement with removal of friable granulation tissue rather than formal surgical excision. At the final follow-up visit, conducted 12 weeks after complete epithelialization, the patient demonstrated full active and passive range of motion of the affected digits without evidence of flexion contracture, sensory deficit, or recurrence.

## Discussion

This case illustrates the successful conservative management of pediatric PG wounds with a topical corticosteroid-antibiotic combination and staged debridement, achieving complete healing without developing a flexion contracture. We are noting an increasing body of evidence for more conservative approaches in select pediatric cases, as noninvasive approaches are gaining popularity. However, the nuances of histopathological verification and contracture prevention in chronic digital neoplasms deserve greater attention.

### Evidence supporting the use of corticosteroid creams in PG in children

Recent studies support topical corticosteroid therapy as a viable first-line treatment for pediatric PG, which aligns with the case presentation of our 7-year-old patient. Barry et al. stated that clobetasol is the most commonly used treatment for pediatric PG.^7^ Additionally, approximately half of the patients showed resolution of their lesions after treatment. This is particularly relevant because treatments tend to be more successful in younger patients (*p* = 0.013) and smaller lesions (*p* = 0.006).^7^ The use of corticosteroids in combination with fusidic acid in our case contributes to the existing body of knowledge. Although there is a lack of literature specifically addressing cortisone-fusidic acid combinations, their anti-inflammatory and antimicrobial activities offer therapeutic advantages. Moustafa et al. reported full recovery using topical clobetasol in a pediatric patient who could not undergo biopsy.^8^ This illustrates the need for minimalistic treatment methods in children, as many find it difficult to cooperate during invasive procedures. This is most likely due to the inhibition of vascular endothelial growth factor (VEGF) and granulation tissue angiogenesis, mimicking the effects of corticosteroids on infantile hemangiomas.^7^

### Comparative effectiveness with other treatment modalities

We acknowledge that the intervention represents a form of surgical management with secondary intention healing; however, we emphasize its staged, limited nature and functional focus rather than categorizing it as formal excisional surgery. A thorough survey of over 1162 cases and 19 different treatment methods revealed that cryotherapy had the lowest overall recurrence rate (1.62%), followed by surgical excision of the lesion with primary closure (2.94%).^9^ Nevertheless, for the treating physician, topical treatments offer significant benefits for children, including the absence of the need for general anesthesia, no risk of scarring, and the possibility of application by the patient at home.

However, the 50% success rate of topical corticosteroids must be considered in the context of a pediatric framework.^7^ Shave excision combined with electrocautery is a common technique; however, its recurrence rates range from 8% to 43.5%, depending on the method and thoroughness of the excision.^7^^,^^10^ Specialized equipment is required for pulsed dye laser treatment, and sometimes multiple sessions are necessary, which achieves an 89% success rate.^11^ Timolol, a topical beta-blocker, has been shown to have results comparable to corticosteroids, resolving cases in 21 days to 3 months. In contrast, a 4% propranolol gel has a 72% response rate.^12^^,^^13^

### Joint and contracture preservation considerations

Flexion contracture prevention is crucial for digital lesions adjacent to the proximal interphalangeal joints. Early mobilization and positioning are helpful and effective in preventing contractures in pediatric hand injury patients. If a person remains in a static position, there is a risk of contracture, which can result in up to 40% muscle loss and shortening of muscle fibers.^14^ Our method of staged debridement, which maintained some joint movement, provided immediate post-injury repositioning, along with gentle range-of-motion exercises.

It is noteworthy that evidence demonstrating preserved wound healing with short-term corticosteroid exposure refers specifically to systemic administration, whereas prolonged systemic therapy is associated with increased wound complications. These findings cannot be directly extrapolated to topical corticosteroids; however, they provide indirect reassurance that brief corticosteroid exposure is unlikely to markedly impair healing.^15^ This chronological difference supports the rationale for our short-duration topical therapy. The rest-with-movement balance approach, combined with prompt mobilization, leverages children’s remarkable healing ability, which research indicates warrants more aggressive rehabilitation than adults.^16^ The absence of contracture in our case corroborates the approach for digital PG, where functional joint preservation is vital.

### Difficulties in managing PG in children close to joints

Children’s fingers pose difficulties because of the digital to periungual regions, where PGs have a more pronounced tendency to bleed and a higher potential for functional impairment.^17^ However, recurrence and complication rates are high because of incomplete excision in cases involving the joints.^17^^,^^18^ The conservative, function-preserving approach used in this case minimized the risk of joint stiffness while avoiding the morbidity associated with formal excisional surgery.

Moreover, children under the age of six have noninvasive approaches tailored to their age group. Evidence suggests that this has a powerful impact on the youngest child demographic.^17^^,^^19^ The mental effect of the procedure, difficulty in complying with postoperative regulations, and increased healing capabilities in children all add to the case for conservative strategies.^17^ The staged debridement technique we implemented allowed for a more limited but conservative, functional, and healthy tissue-sparing autotomy, in line with the principles of pediatric hand surgery regarding tissue preservation in children. Furthermore, regardless of the chosen therapeutic strategy, follow-up is essential to monitor recurrence and manage any complications. Regular check-ups and patient education on the signs of recurrence are crucial aspects of post-treatment care.^20^^,^^21^

### Limitations

The lack of tissue samples is perhaps our greatest limitation. Current best practices suggest that all excised PG specimens should undergo histopathological examination to rule out mimicking malignancies, primarily amelanotic melanoma, which is the most significant differential diagnosis of PG. Studies have shown that 3.0% of clinically considered benign skin lesions are found to be pre-malignant or malignant upon histopathological evaluation, with family physicians demonstrating only 38% sensitivity to malignancy.^17^^,^^22^

The option of proceeding without a tissue diagnosis involves balancing many competing concerns. While most experts agree that PG is “usually a clinical diagnosis,” consensus states, “when excision is performed, specimens must always be sent for histopathologic assessment to rule out red-flag diagnoses.” In our case with the classical appearance and response to treatment, it seemed defeasible in terms of flexible reasoning, albeit with undoubted diagnostic and legal risks. The strong response to treatment supports the retrospective diagnosis but does not eliminate the risk of overlooking atypical vascular lesions.

### Clinical implications and future research

**Figure 1:**
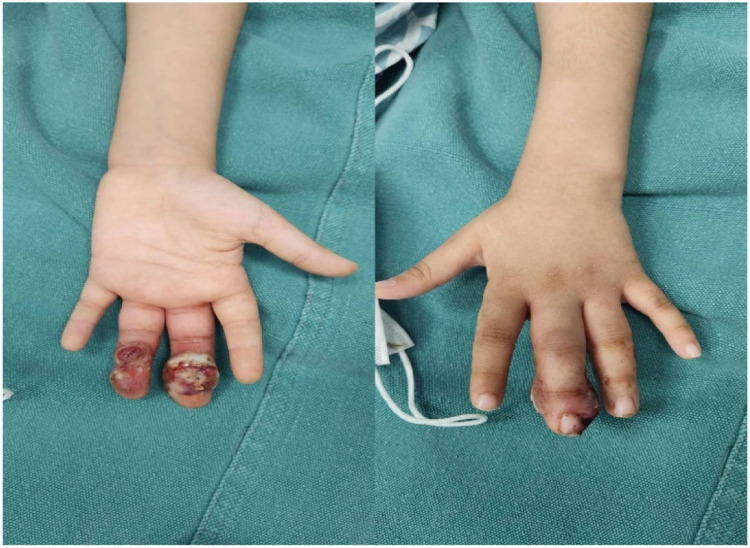
Initial presentation. Volar swelling and erythematous granulation tissue of the left middle and ring fingers at first presentation.

Our case adds to the growing body of evidence supporting less conservative management strategies for children with PG. For patients with small lesions (<5 mm) that do not bleed, we recommend a stepwise approach starting with topical corticosteroid therapy (clobetasol 0.05% twice daily under occlusion for 4–6 weeks), particularly in younger patients. The addition of topical antibiotics could be helpful, especially for lesions complicated by secondary infections or crusting. Staged debridement can address bulk disease while preserving function, which is a highly desirable attribute for digits. Figure 1.

**Figure 2:**
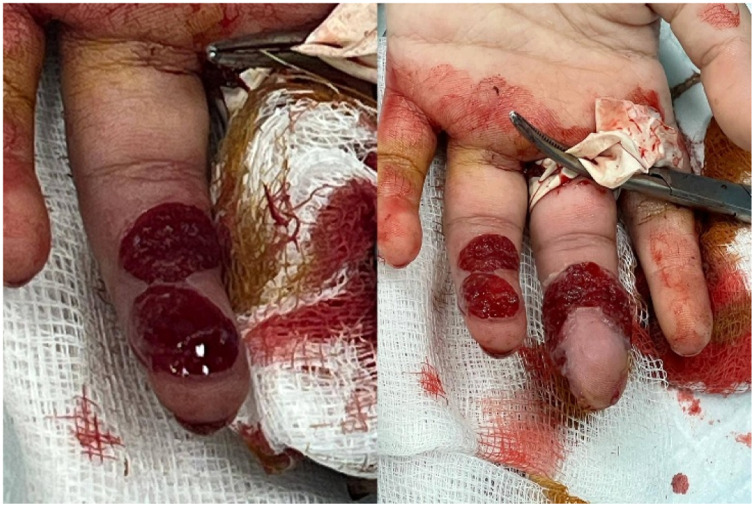
Debridement findings. Intraoperative appearance showing lobular granulation tissue and removal of embedded sutures during staged bedside debridement.

Key areas for future research focus on the expanding role of telemedicine in monitoring treatment response and remote patient evaluations. Both are critical, given the reduced need to access healthcare facilities. Furthermore, with digital photography, patients can submit photographs of their lesions, allowing for the remote assessment of treatment progress. This could decrease the number of clinic visits while maintaining treatment oversight Figure 2.

## Conclusions

**Figure 3:**
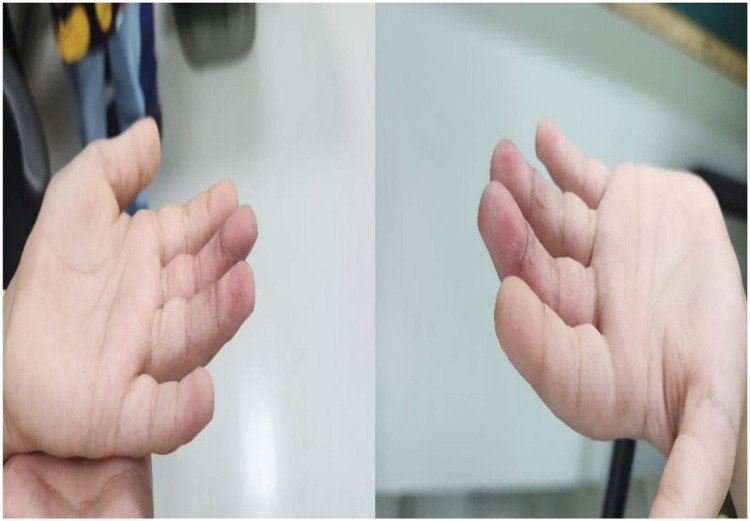
Post-treatment outcome. Marked improvement with epithelialization and full range of motion following corticosteroid–fusidic acid dressings.

This case illustrates that the use of topical corticosteroid-antibiotic therapy in conjunction with staged debridement is effective in managing pediatric digital PG without resulting in contracture formation. Although surgical excision remains the definitive treatment, this illustrates the value of conservative approaches, especially for selected patients, such as very young children with small lesions, in whom surgical morbidity is a concern. The lack of histopathological verification is a significant limitation that affects the clinical rationale and needs to be addressed. Supporting these findings, diagnostic precision emphasizes tailored strategies and customizable approaches that focus on functional outcomes and prioritize bolstered options, thereby avoiding intervention frameworks. Optimizing the direction of pediatric PG treatment requires prospective work based on standard conservative treatment techniques, systematically incorporating guided histopathology Figure 3.

## Funding

None.

## Ethics approval

Not required.

## Informed consent

Written informed consent for publication of the case details and clinical images was obtained from the patient’s legal guardian.

## Author contributions

All authors contributed to the conceptualization, drafting, and revision of the manuscript and approved the final version.

## Declaration of competing interests

None.
